# Perioperative symptomatic venous thromboembolism after immediate chemoprophylaxis in patients with pelvic and lower-extremity fractures

**DOI:** 10.1038/s41598-020-62333-z

**Published:** 2020-03-25

**Authors:** Jin Kyu Lee, Ja Wook Koo, Soo-Young Jeong, Sihoon Choi, Ki-Chul Park, Kyu-Tae Hwang

**Affiliations:** 1 Department of Orthopedic Surgery, Hanyang University Hospital, Seongdong-gu Seoul, Republic of Korea; 2 Department of Orthopedic Surgery, Hanyang University Guri Hospital, Guri, Gyeonggi-do Republic of Korea

**Keywords:** Fracture repair, Risk factors

## Abstract

The purpose of this study was to investigate the incidence of symptomatic venous thromboembolism (VTE) after chemoprophylaxis in patients with pelvic and lower-extremity fractures, and to identify risk factors for VTEs in this subgroup of patients. To detect VTE, multi-detector computed tomography (CT) angiography was performed. Of 363 patients assessed, the incidence of symptomatic VTE was 12.4% (45 patients), and the incidence of symptomatic PE was 5.2% (19 patients). For the risk-factor analysis, a higher Charlson comorbidity index (p = 0.037), and a history of external fixator application (p = 0.007) were associated with increased VTE risk. Among patients who had VTE, male sex (p = 0.017), and above-the-knee fractures (p = 0.035) were associated with increased pulmonary embolism (PE) risk. In conclusions, the incidence of VTE in post-traumatic patients is not low after chemoprophylaxis. Risk factors for VTE and PE are different among patients with pelvic and lower-extremity fractures.

## Introduction

Deep vein thrombosis (DVT) and pulmonary embolism (PE), known as venous thromboembolism (VTE), occur every year in approximately 900,000 people in the United States, with a death toll of 300,000^1^. Patients with severe trauma are at risk of VTE, and in 25% of severely traumatised patients, coagulopathy occurs immediately after injury, resulting in a 5-fold increase in mortality^2–6^. The reported incidence of post-traumatic VTE ranges between 2.6% and 63% depending on factors including patients’ characteristics (e.g., injury severity and injury pattern), type of VTE prevention used, sensitivity of the screening test, and other factors^7–10^. Prophylaxis of VTE is based mainly on physical therapy and chemotherapy^11–14^, and chemotherapy is more efficacious than physical therapy in terms of lowering the incidence of VTE^15^. Most studies of the incidence of VTE in post-traumatic patients include various patterns and severity of trauma, and it is difficult to estimate the true incidence of VTE in specific types of fractures or provide an optimal guideline for prophylaxis of VTE in this subgroup of patients^16–18^. Furthermore, identification of risk factors has proven useful in defining high-risk patients and corresponding prophylaxis appears mandatory for all trauma patients.

The purpose of this study was to investigate the incidence of symptomatic VTE after chemoprophylaxis in patients with pelvic and lower-extremity fractures. Multivariate logistic regression was also performed to identify risk factors for VTEs in this subgroup of patients.

## Results

Of 363 patients assessed, 212 were male and 151 were female, with an average age of 56 years (range, 21–73 years). Demographic and clinical data including patients’ comorbidities and Charlson index values are presented in Table 1. There were significantly higher proportion of patients with a history of peripheral vascular disease in patients who had VTE compared to patients without VTE (31.1% vs 1.3%, p < 0.001). There were 28 cases of acetabulum fracture, 17 cases of pelvic bone fracture, 174 cases of femur fracture, 137 cases of tibia fracture, and 75 cases of ankle fracture (Table 2). Sixty-five patients had multiple fractures involving 2 or more anatomical locations. Forty-five patients (12.4%) were diagnosed with symptomatic VTE. DVT and PE occurred in 34 (9.4%) and 19 (5.2%) patients, and 8 patients (2.2%) were diagnosed with both DVT and PE. Twelve patients (3.3%) were diagnosed with VTE before definitive fixation surgery, and 3 of those were diagnosed as pulmonary embolism. These patients developed VTE on average 6.3 ± 8.6 days from the date of injury. Thirty-three patients (9.1%) were diagnosed with a VTE that occurred after definitive fixation surgery, with the events occurring an average of 6.6 ± 7.6 days after surgery, and 16 of those were diagnosed as pulmonary embolism. Thirteen patients (3.6%) were diagnosed with distal DVT, and 3 of those coexisted with PE. Thirteen patients (3.6%) were also diagnosed with proximal DVT, and 3 of those coexisted with PE. Eight patients (2.2%) were diagnosed to have both proximal and distal DVT in the same lower extremity, and 2 of those coexisted with PE.

**Table 1:** Baseline demographics of the patients without and with venous thromboembolism (VTE).

Variables	VTE (**−**) (N = 318)	VTE (+) (N = 45)	*P*-value
Age	54(21–71)	58(24–73)	0.228
Body mass index	23.53(21.19–26.04)	24.01(21.15–25.89)	0.575
Sex			
Male	191(60.1)	21(46.7)	0.088
Female	127(39.9)	24(53.3)	
Charlson comorbidity index	1(0–3)	2(1–3)	**0.036**
Myocardial infarction	8(2.5)	1(2.2)	1.000
Congestive heart failure	5(1.6)	1(2.2)	0.551
Peripheral vascular disease	4(1.3)	14(31.1)	**<0.0001**
Cerebrovascular disease	25(7.9)	5(11.1)	0.398
Dementia	11(3.5)	0(0.0)	0.372
Chronic pulmonary disease	2(0.6)	0(0.0)	1.000
Connective tissue disease	14(4.4)	3(6.7)	0.454
Ulcer disease	1(0.3)	1(2.2)	0.233
Mild liver disease	5(1.6)	0(0.0)	1.000
Liver cirrhosis	3(0.9)	0(0.0)	1.000
Diabetes without end-organ damage	55(17.3)	10(22.2)	0.420
Diabetes with end-organ damage	3(0.9)	0(0.0)	1.000
Chronic kidney disease or dialysis	4(1.3)	0(0.0)	1.000
Tumor-localized	16(5.0)	2(4.4)	1.000
Tumor-Metastasis	1(0.3)	0(0.0)	1.000
Leukemia	0(0.0)	1(2.2)	0.124

Non-normally distributed numerical variables are presented by median (Q1–Q3) and tested by Wilcoxon rank-sum test, and categorical variables are presented by N (%) and tested by chi-squared test or Fisher’s exact test.

**Table 2:** Fracture location according to the anatomy of the bone involved.

Fracture location	N	%
Acetabulum	28	6.5
Pelvis	17	3.9
Femur neck	19	4.4
Femur Intertrochanteric	54	12.5
Femur subtrochanteric	10	2.3
Femur shaft	60	13.9
Distal femur	31	7.2
Proximal tibia	42	9.7
Tibia shaft	34	7.9
Distal tibia	61	14.2
Ankle	75	17.4
Total	431	100.0

**Figure 1:**
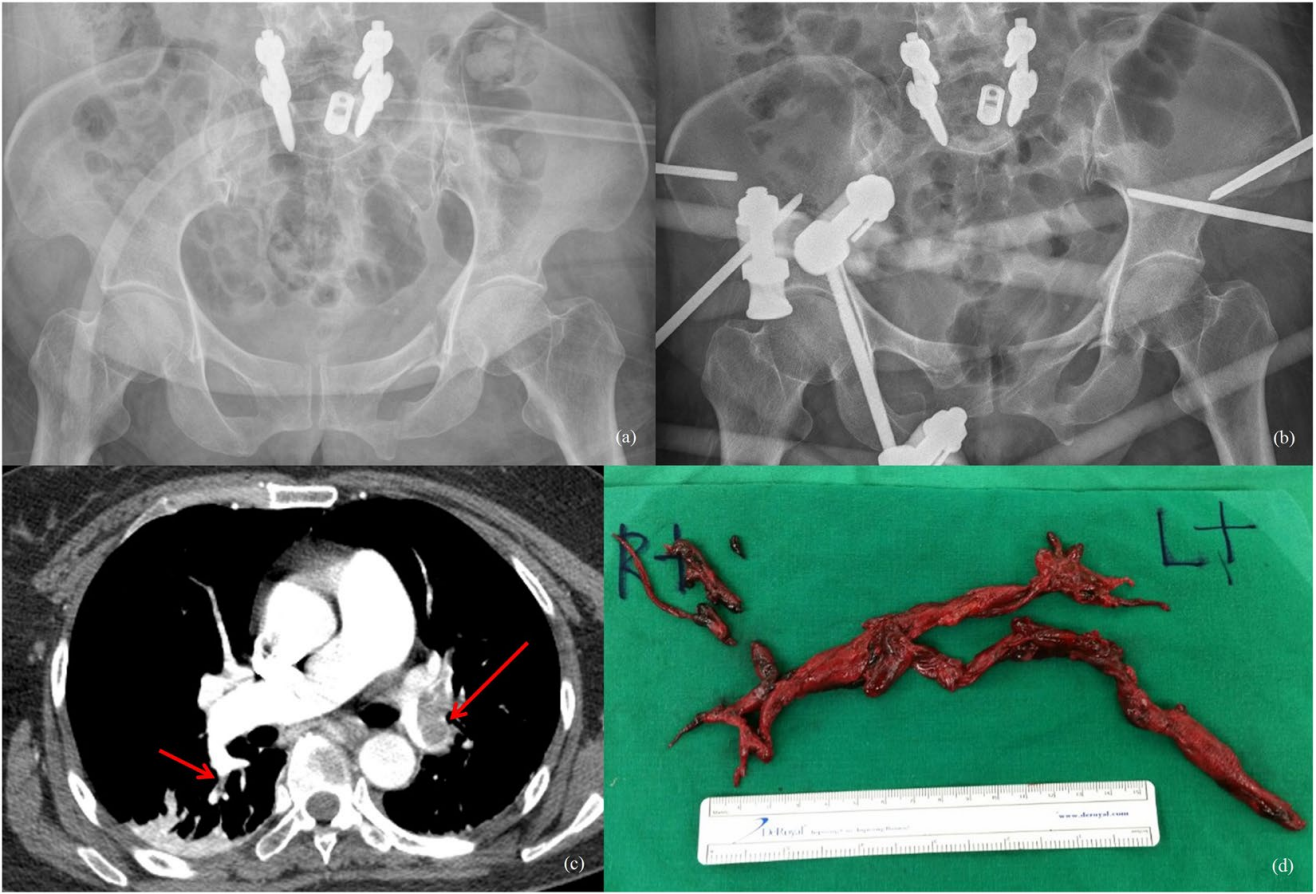
A 52-year-old female patient with a left rami fracture and diastasis of the left SI joint after a traffic accident underwent general anaesthesia for pelvic ring stabilization (**a**). After general anaesthesia, the patient rapidly developed hypoxia and tachycardia and the operation was not performed. Only external fixation was performed (**b**). CT angiography was performed to confirm total occlusion of the left pulmonary artery and right segmental thrombus (red arrow) (**c**). Thrombectomy was immediately performed (**d**).

For treatment, either Enoxaparin or Dalteparin via subcutaneous injection at treatment dose was used for 5–7 days and oral medication (e.g. dabigatran) was continued for 3 months. In a single case of PE, an emergency thrombectomy was required. (Fig. 1).

None of the patients suffered from wound complications requiring hematoma evacuation, cessation of pharmacologic agents, or surgical management. Furthermore, none of the patients suffered from major bleeding complication defined as any intracranial, intraocular, retroperitoneal, intraspinal, or pericardial bleeding^19^.

Univariate analysis showed that a history of external fixator application (p = 0.020) and open fractures (p = 0.011) were associated with increased VTE risk in this study. After stepwise multivariable regression analysis, a higher Charlson comorbidity index (p = 0.037) and a history of external fixator application (p = 0.007) were associated with increased VTE risk (Table 3). Subgroup analysis within patients who had VTE (e.g., patients with PE versus VTE patients without PE) revealed that male sex (p = 0.017) and above-the-knee fracture (p = 0.035) were associated with increased PE risk (Table 4). However, the location of the DVT was not significantly correlated with occurrence of PE (p = 1.000).

**Table 3:** The association of demographic and clinical variables on occurrence of venous thromboembolism (VTE) assessed by logistic regression analysis.

Variables	Univariable			Multivariable		
	Odds ratio	95% C.I.	*P*-value	Odds ratio	95% C.I.	*P*-value
Age	1.01	0.99–1.03	0.214			
Body mass index	1.01	0.96–1.08	0.655			
Sex (Female)	1.72	0.92–3.22	0.091			
Multiple fracture (vs single)	1.53	0.71–3.29	0.275			
Injury severity score (>8)	1.68	0.46–6.13	0.435			
Below knee fracture (vs above knee fracture)	1.33	0.69–2.55	0.399			
Charlson comorbidity index	1.13	0.98–1.30	0.102	1.18	1.01–1.38	**0.037**
External fixation (yes)	2.34	1.15–4.79	**0.020**	2.98	1.34–6.60	**0.007**
Open (vs closed)	2.53	1.23–5.19	**0.011**			
Flap (yes)	2.96	0.79–11.09	0.107			
Gustilo classification grade						
1	<0.001	<0.001->999.999	0.962			
2	0.29	0.06–1.50	0.140			
3	Ref.

**Table 4:** The association of demographic and clinical variables on occurrence of pulmonary embolism (PE) among patients with venous thromboembolism (VTE) assessed by logistic regression analysis.

Variables	Univariable			Multivariable		
	Odds ratio	95% C.I.	*P*-value	Odds ratio	95% C.I.	*P*-value
Age	1.00	0.97–1.03	0.924			
Body mass index	1.01	0.89–1.15	0.853			
Sex (Female)	0.21	0.06–0.74	**0.015**	0.16	0.03–0.71	**0.017**
Open fracture (vs closed)	0.50	0.13–1.98	0.326			
Multiple fracture (vs single)	2.54	0.60–10.70	0.205			
Injury severity score (>8)	2.94	0.25–35.06	0.394			
Below knee fracture (vs above knee fracture)	0.27	0.07–1.01	0.052	0.20	0.04–0.89	**0.035**
Charlson comorbidity index	0.92	0.67–1.26	0.606			
External fixation (yes)	0.50	0.13–1.98	0.326			

## Discussion

The present study was conducted to investigate the incidence of symptomatic VTE after receiving chemoprophylaxis in patients who underwent surgery for pelvic and lower-extremity fractures, and to identify the risk factors for VTE occurrence in this subgroup of patients. In this study, the incidence of symptomatic VTE detected by CT angiography was 12.4% (45 out of 363 patients), and the incidence of symptomatic PE was 5.2% (19 out of 363 patients). For the risk-factor analysis, a higher Charlson comorbidity index and a history of external fixator application were associated with increased VTE risk. Among patients who had VTE, male sex, and above-the-knee fractures were associated with increased PE risk.

Although the reported incidences of symptomatic and fatal thromboembolic events are low, the risk of symptomatic VTE has changed little over the past 20 years^20,21^. There is a general agreement that prophylaxis against VTE is necessary in post-traumatic patients, but a tendency to underestimate this complication persists, as the majority of patients with small or asymptomatic VTEs do not come under clinical suspicion and are therefore never evaluated^22^.

It has been recommended that post-traumatic patients receive pharmacologic prophylaxis and/or be treated with an intermittent pneumatic compressive device, rather than receive no prophylaxis^11–15^. A gold standard for prophylaxis has yet to be identified. In a study by Stannard *et al*., although the incidence of VTE following chemotherapy alone was 13.4%, the incidence fell further to 8.7% when both drugs and mechanical methods were used^23^. However, application of a pneumatic compressive device in patients with corresponding lower extremity fractures may not be possible. Traditionally, orthopaedic surgeons have been concerned about the effects of VTE prophylaxis on perioperative bleeding, which can result in hematoma, infection and wound problems. There has also been a concern that patients at low risk of developing symptomatic VTE may receive excessive anticoagulation^24^. Accordingly, updated guidelines from both the American Academy of Orthopaedic Surgeons and the American College of Clinical Pharmacy have focused more on the safety of prophylactic agents and symptomatic and fatal VTE events^25,26^.

The reported incidence of post-traumatic VTE ranges between 2.6% and 63%, depending on the demographics of the study population, the nature of the injuries, type of prophylaxis used, and method to detect the lesions^7–10^. Abelseth *et al*. studied the incidence of DVT in 176 patients who had undergone operative fixation for fractures of the lower extremity distal to the hip and reported a 28% incidence of DVT (mostly distal asymptomatic DVTs) detected by screening for venography^16^. Goel *et al*. performed a randomised controlled trial of treating 238 patients who had undergone operative fixation for fractures of below-the-knee lower extremity with placebo or low-molecular-weight heparin (LMWH) and reported no significant difference in the incidence of DVT (8.7% in LMWH group vs 12.6% in placebo group) as detected by screening venography^17^. Niikura *et al*. studied the rate of VTE after complex lower-extremity fracture surgery consisting of temporary external fixator application followed by definitive internal fixation, and found a high incidence of VTE (38.5%, 15 out of 39 patients) was detected by contrast-enhanced CT without pharmacological prophylaxis, although no PEs were fatal^18^. In the present study, pelvic and lower-extremity fractures were assessed for symptomatic VTEs. The incidence of symptomatic VTE detected by CT angiography was 12.4% (45 out of 363 patients) and the incidence of symptomatic PE was 5.2% (19 out of 363 patients) with pharmacological prophylaxis applied. Although not directly comparable, the incidence of symptomatic VTE in the present study was high in post-traumatic patients with lower-extremity fractures, even with pharmacological prophylaxis.

Knudson *et al*. identified 450,375 trauma patients and the risk factors associated with development of VTE^27^. They reported an age of over 40 years, a lower-extremity fracture more than 3 points in abbreviated injury scale (AIS), applying a ventilator for more than 3 days, a head injury more than 3 points in AIS, venous injury, and cases requiring major operative procedure as risk factors. Wahlsten *et al*. studied 57,619 trauma patients and found that the incidence of VTE was low following surgery for fractures distal to the knee, but the risk increased with the presence of risk factors including use of oral contraception by patients eighteen to fifty years of age, previous DVT, previous PE, coagulopathy, and peripheral artery disease^28^. Furthermore, Thorson *et al*. prescreened 534 trauma patients with a risk assessment profile, and reported that high-risk profile score and pelvic fracture with prolonged intervention are independent predictors for VTE development, despite thromboprophylaxis^29^. However, few studies have reported the association between VTE incidence and patient factors such as sex, BMI, and underlying disease in trauma patients with fractures. In the present study, a higher Charlson comorbidity index and a history of external fixator application were associated with increased VTE risk. Among conditions for Charlson comorbidity index scoring, there were significantly higher proportion of patients with a history of peripheral vascular disease in patients who had VTE compared to patients without VTE (31.3% vs 1.3%, p < 0.001), and this may have influenced the results. Among patients who had VTE, male sex, and above-the-knee fractures were associated with increased PE risk. It is noteworthy that patients who had external fixator application were associated with increased VTE risk, and a longer period of immobilization would have contributed to thrombus formation in the vessels^4^. For the PE risk, up to 40% of proximal DVTs have an association with PE, and above-the-knee fractures would have caused more injury to the proximal vein, leading to formation and emboli of the thrombus at a more proximal area^30^. It was surprising to find being male as a risk factor for PE. Several studies have assessed the relationship between sex and incidence of VTE, and found the VTE recurrence rate is consistently higher in men, suggesting that higher prevalence of PE-associated proximal DVT in men partly accounts for the higher VTE recurrence rate in men^31,32^. It is possible that the higher prevalence of PE-associated proximal DVT in men, as with above-the-knee fractures being a risk factor for PE, is associated with PE risk in patients with pelvic and lower-extremity fractures.

This study has several limitations. First, two different LMWHs were used as there was institutional formulary change in LMWH prophylaxis from Dalteparin to Enoxaparin. However, as effectiveness of Dalteparin and Enoxaparin for thromboprophylaxis after traumatic injury has been reported to be similar by numerous studies^33–35^, the effect of differing LMWH prophylaxis on VTE occurrence should have been minimal. In a study that included 5880 trauma patients, the VTE rate was 3.3/1000 days in the Enoxaparin period vs 3.8/1000 days in Dalteparin period proving similar prophylactic effect of both LMWHs in trauma patients^33^. Second, only patients eligible to use pharmacological anti-coagulation prophylaxis were enrolled. Patients with co-existing head, chest, or abdominal trauma requiring treatment were excluded from the study. The findings in our study cannot be generalised to severely traumatised patients with a high ISS or those requiring intensive-care unit hospitalization. Third, as this was a single-institution study, it may not reflect universal practices, and the number of patients assessed was relatively small, accounting for the low incidence of PE. Fourth, symptoms of VTE were subjectively assessed by the senior surgeon, and selection bias issue may be an issue. Fifth, it is not possible to directly compare the results between studies due to inhomogeneity in study populations, VTE prophylaxis, and detection methods. Finally, other factors including use of oral contraception, timing of weight bearing, and ankle mobilization were not thoroughly assessed.

Despite pharmacological prophylaxis, the incidence of symptomatic VTE detected by CT angiography was 12.4% (45 out of 363 patients), and the incidence of symptomatic PE was 5.2% (19 of 363 patients). A higher Charlson comorbidity index and a history of external fixator application were associated with increased VTE risk. Male sex, and above-the-knee fracture were associated with increased PE risk. In such high-risk patients close monitoring with more aggressive prophylactic regimens may be beneficial.

## Materials and Methods

### Study population

The Institutional Review Board of Hanyang University hospital approved the study, and all patients provided informed consent. All procedures performed in this study involving human participants were in accordance with ethical standards of the institutional and/or national research committee and with the 1964 Helsinki Declaration and its later amendments or comparable ethical standards. Between March 2012 and July 2017, 392 patients who underwent surgery for acute pelvic bone and lower extremity fractures were assessed retrospectively. Exclusion criteria included a history of thromboembolic disease; the use of anticoagulants; renal insufficiency; liver cirrhosis and hepatitis; a history of haemorrhagic disease; co-existing brain, thoracic or abdominal trauma requiring treatment; combined upper extremity fractures; and simple lower-extremity fractures involving foot or patella. A total of 363 patients were enrolled in the study. All patients underwent pharmacologic treatment immediately after admission (within 12 hours of initial presentation) to prevent VTE and treatment continued until partial weighted crutch walking or active range of motion on injured lower extremity was possible. Patients received LMWH, either Enoxaparin or Dalteparin, via subcutaneous injection at a dose of 40 mg/day and 5000 units/day, respectively. Two different LMWH formulations were used as there was institutional formulary change in LMWH prophylaxis from Dalteparin to Enoxaparin in 2015.

**Figure 2:**
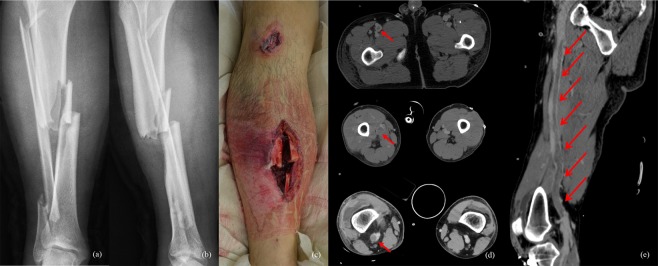
A 41-year-old male patient with right tibio-fibular open fracture and left ankle fracture after a motorcycle accident developed right lower-extremity severe swelling and pain during passive ankle and toe motion (**a–c**). CT angiography was performed. A massive thrombus in the right common femoral vein and calf were confirmed (red arrow) and chemical treatment was administered continuously (**d**,**e**).

Patients were not routinely screened for VTE, and only those with symptoms or signs suggestive of VTE were assessed for definitive diagnosis. Clinical signs suggestive of symptomatic DVT, including pain and marked swelling of the corresponding calf or thigh, tenderness on indurated venous segment, Homan’s sign, variable skin discoloration of the lower extremity, and prominence of superficial veins were evaluated daily^36^. PE, defined as symptomatic when a patient had symptoms such as dyspnoea, tachypnoea, chest pain, and cough, was suspected with sudden hypoxia (O_2_ saturation <95%) or hypoxemia (PaO_2_ < 83 mmHg)^37^. To detect VTE, 64-channel multidetector computed tomography (Brilliance 64®, Philips, Eindhoven, the Netherlands) was performed (Fig. 2).

To evaluate risk factors for VTE, demographic and clinical data were collected from medical records (Table 1). Fracture location was defined according to the anatomy of the bone involved (e.g., acetabulum, pelvis, femur, tibia, or ankle) (Table 2). Multiple fractures were defined as fractures involving at least 2 locations. Because DVT in the pelvis and lower extremities proximal to the popliteal artery is the most frequent source of emboli in the pulmonary artery^30^, fracture location was further classified as proximal to the fracture in an area proximal to a knee joint (e.g., acetabulum, pelvis, and femur) and as distal when the fracture was limited to an area distal to a knee joint (e.g., tibia and ankle). The injury severity score (ISS) scale was used to represent the severity of the injuries, which were categorised as mild (<9), moderate (9–15), or severe (16–25)^38^. Gustilo open-fracture classification was used to grade open fractures^39^. The Charlson comorbidity index^40^ which contains 19 issues including diabetes with diabetic complications, congestive heart failure, peripheral vascular disease (intermittent claudication, prior bypass for chronic arterial insufficiency, history of gangrene, acute arterial insufficiency, untreated aneurysm >6 cm), chronic pulmonary disease, liver disease, hemiplegia, renal disease, leukemia, lymphoma, and metastatic tumor was used to categorize comorbidities of patients (Table 1).

### Statistical analysis

Statistical significance was tested using a Wilcoxon rank-sum test, Pearson’s chi-squared test, and Fisher’s exact test as appropriate. Non-normally distributed numerical variables were tested by Wilcoxon rank-sum test, and categorical variables were tested by either chi-squared test or Fisher’s exact test. Factors including sex, age, body mass index (BMI), Charlson comorbidity index, and injury-related variables (single versus multiple fracture, proximal versus distal fracture, ISS, and severity of open fracture) were assessed with logistic regression analysis. Stepwise linear regression analysis was performed and a model was selected based on the value of Akaike’s Information Criterion. Statistical significance was set at 0.05 and SAS version 9.4 (SAS Institute Inc., Cary, NC, USA) and R package version 3.5.1 were used for all analyses.

## Supplementary information

Supplementary dataset.

## Data Availability

All data analysed during this study are available from the corresponding author on reasonable request.
